# Tumor-infiltrating CD8+ T cells combined with tumor-associated CD68+ macrophages predict postoperative prognosis and adjuvant chemotherapy benefit in resected gastric cancer

**DOI:** 10.1186/s12885-019-6089-z

**Published:** 2019-09-14

**Authors:** Jun Lu, Yu Xu, Yuan Wu, Xiao-yan Huang, Jian-wei Xie, Jia-bin Wang, Jian-xian Lin, Ping Li, Chao-hui Zheng, Ai-min Huang, Chang-ming Huang

**Affiliations:** 1 Department of Gastric Surgery, Fujian Medical University Union Hospital, Fuzhou, China; 2 Department of General Surgery, Fujian Medical University Union Hospital, Fuzhou, China; 3 Key Laboratory of Ministry of Education of Gastrointestinal Cancer, Fujian Medical University, Fuzhou, China; 4 Department of Pathology, the School of Basic Medical Sciences, Fujian Medical University, Fuzhou, China; 5 lnstitue of Oncology of Fujian Medical University, Fuzhou, China

**Keywords:** Adjuvant chemotherapy benefit, Gastric cancer, Prognosis, Tumor-infiltrating neutrophils, Tumor-associated macrophages

## Abstract

**Background:**

Tumor-infiltrating immune cells are present in various malignant tumors, but their clinical significance in gastric cancer (GC) remains unclear. This study aimed to investigate the prognostic significance of tumor-infiltrating lymphocytes (TILs) and tumor-associated macrophages (TAMs).

**Methods:**

Using a prospective database containing 401 cases of GC, we evaluated TIL (cluster of differentiation 8 (CD8) expression) and TAM (cluster of differentiation 68 (CD68) expression) statuses via immunohistochemical staining.

**Results:**

Compared with CD8+ TIL-negative cases (*n* = 196, 48.6%), CD8+ TIL-positive cases (*n* = 205, 51.1%) showed significantly better recurrence-free survival (RFS) [log-rank *p*<0.001; multivariate HR: 0.372; 95% confidence interval (CI): 0.239–0.579, *p*<0.001]. In contrast, compared with CD68+ TAM-negative cases (*n* = 217, 54.1%), CD68+ TAM-positive cases (*n* = 184, 45.9%) had significantly poor RFS [log-rank *p*<0.001; multivariate HR: 2.182; 95% CI: 1.435–3.318, *p*<0.001]. Thus, patients with a positive CD8+ TIL and negative CD68+ TAM status exhibited significantly increased RFS. Multivariate analysis demonstrated that CD8+ TILs and CD68+ TAMs may serve as independent prognostic markers for RFS. Incorporating CD8+ TIL and CD68+ TAM statuses into the AJCC TNM system generated a predictive model with better predictive accuracy for RFS. More importantly, patients with a positive TIL and negative TAM status showed a tendency of improved RFS after postoperative adjuvant chemotherapy (PAC). Similar results were obtained by overall survival (OS) analysis.

**Conclusions:**

CD8+ TIL and CD68+ TAM statuses were identified as independent prognostic factors that may be integrated into the current TNM staging system to refine risk stratification and to better predict the survival benefit from PAC in patients with GC.

**Trial registration:**

The current controlled trial was registered at ClinicalTrials.gov (ID: NCT02327481) on December 30, 2014.

**Electronic supplementary material:**

The online version of this article (10.1186/s12885-019-6089-z) contains supplementary material, which is available to authorized users.

## Background

Gastric cancer (GC) is one of the most common malignant tumors in both sexes worldwide [1]. Regarding advanced GC, it has been suggested that a PAC regimen based on 5-fluorouracil (5-Fu) should be used as a first-line postoperative therapy [2–6]. However, the response to PAC varies among patients. Of greater concern is the lack of a reliable criterion for identifying patients at high risk of recurrence, which in turn makes it difficult to distinguish between patients who will benefit from PAC when considering the survival rate after surgery [6–8]. Accordingly, there is considerable interest in exploring the potential benefits of PAC for GC patients.

In recent decades, convincing evidence has emerged that the tumor microenvironment (TME) and inflammation play a key role in the development of many malignant tumors, including GC [1, 9, 10]. Tumor-infiltrating lymphocytes (TILs) and tumor-associated macrophages (TAMs) are two main components of the TME that have shown prognostic value in previous studies [11, 12]. Therefore, incorporating these immunological parameters into the established TNM staging system may increase the prognostic value for further stratification and better management of patients with different prognoses.

Regardless, little is known about the prognostic significance of TILs combined with TAMs in GC, and the impact of these cells on PAC benefit in GC remains unclear. In this study, we evaluated TILs and TAMs in more than 400 GC patients by immunohistochemical staining of CD8 and CD68. In addition, we used subgroup analysis to explore the relationship between TILs/TAMs and survival rates as well as the benefit of PAC based on 5-FU for survival. The results of this study will clarify the important prognostic role of lymphocyte and macrophage infiltration in GC and help to redefine subgroups of patients who are likely to benefit from PAC.

## Methods

### Patients and specimens

From January 1, 2015, to April 1, 2016, 438 patients were recruited from Union Medical College Hospital of Fujian Medical University for a randomized clinical trial. The final analysis included 419 patients (clinicaltrials.gov No. NCT02327481). Detailed information on the selection, exclusion, quality control and randomization has been reported previously [13, 14]. This study is a sub-study of the above clinical trials. After excluding 10 patients with neuroendocrine cancer, 6 patients with palliative surgery and 2 patients without GC evidence, this analysis involved 401 patients who underwent therapeutic gastrectomy and pathologically confirmed as having stage I, II, or III gastric adenocarcinoma (pT1-4aN0-3 M0) according to the 7th American Joint Committee on Cancer staging [15]. The formalin-fixed paraffin-embedded (FFPE) tissues of the 401 patients with GC were analyzed, with all specimens being independently re-evaluated by two gastrointestinal pathologists. None of the patients had received any anticancer treatment before surgery. PAC treatment is acceptable for patients with advanced or early diagnosis of excessive lymph node metastasis, and PAC was classified as received or not received [6]. A total of 256 patients (63.8%) received at least one 5-FU baseline PAC cycle [8, 16]. According to the Japanese Classification of Gastric Carcinoma, PAC with S-1 is recommended for GC patients with pathological stage II or III disease [17]. In our center, fluoride-assisted chemotherapy is also recommended for most stage II or III GC patients, depending on the patient’s wishes and physical condition [18]. In this cohort, 256 patients (63.8%) received 5-FU adjuvant chemotherapy with a median cycle of 5 (range 1–12), similar to a previous study [19]. Specifically, 76.2% (195/256) received the SOX regimen (S-1 plus oxaliplatin), 12.1% (31/256) the FOLFOX regimen (5-FU + oxaplatin+leucovorin), 9.4% (24/256) the S1 + paclitaxel regimen, and 2.3% (6/256) the S1 + docetaxel regimen. Overall survival (OS) was defined as the interval between the date of surgery and the date of death or last visit. Recurrence-free survival (RFS) was defined as the period from the date of surgery to the date of diagnosis of recurrence. The median follow-up time was 29 months (range 3–41 months).

All patients provided written informed consent before sampling, and the research procedure was approved by the Institutional Review Committee of Union Medical College Hospital of Fujian Medical University.

### Immunohistochemistry (IHC) and evaluation

Formalin-fixed, paraffin-embedded GC surgical specimens were used for IHC, as previously described [20, 21]. Briefly, continuous paraffin slices with a thickness of 4 μm were immersed in xylene and rehydrated by an ethanol series, PBS buffer and deionized water for 5 min each. The slides were heated to 100 °C for 20 min in pH 9 Tris-based solution. All slides were incubated with the primary antibody for 60 min at 37 °C for 1 h (dilutions: rabbit anti-human CD8 monoclonal 1:500, SP16, MAIXIN. BIO, Fuzhou, China; mouse anti-human CD68 monoclonal 1:500, KP1, MAIXIN. BIO, Fuzhou, China) and then washed. As a secondary antibody, mouse IgG was added for 30 min, and the slides were again washed. The sections were processed with the universal SP Elivision-plus kit (kit-9903, MAIXIN. BIO, Fuzhou, China), and the sections were counterstained with hematoxylin.

All specimens were selected by the local pathologist. Two pathologists (Y. X and AM. H) blinded to the tumor clinicopathologic characteristics and patient outcomes independently scored all slides, with discrepancies resolved by consensus. The tissue sections were screened in a low-power field (100×), and the 5 most representative fields were selected using an Olympus BX46 research microscope (Olympus, Tokyo, Japan).

CD8 is mainly distributed on the cell membrane and in cytoplasm. Using high-power microscopy, 5 fields of view with the richest permeability of GC were selected from each slice. The percentage of CD8+ T lymphocytes among total lymphocytes was calculated. The average values of 5 fields were taken as the density (%) of CD8 + TILs. Briefly, TILs were counted separately based on their location in the epithelium or interstitium. T lymphocytes infiltrating into cancer cell nests, designated intraepithelial T lymphocytes, were counted in high-power fields (HPFs) at 200× magnification. Intratumoral T cells were classified as 0, 1, 2, or 3 (≤5, 6 to 19, 20 to 34, or ≥ 35 T cells per high-power field, respectively), and the average count was calculated. Stromal (tumor-associated stroma and/or perivascular spaces) T lymphocytes were evaluated by the same method. To assess the density of tissue-infiltrating CD68+ TAMs, the respective areas of the tumor nest and peritumoral tissue were measured at 200× magnification. The number of nucleated CD68+ TAMs in each area was then counted manually, and the results are expressed as cells per field. Positive-staining cells less than or equal to the size of circulating monocytes (~ 10 μm) were excluded. CD68+ TAMs were scored as 0, 1, 2, or 3 (≤10, 11 to 29, 30 to 49, or ≥ 50 TAMs/ high-power field, respectively). The average number of the two researchers was used in the following analysis to minimize variability. After hematoxylin-eosin staining, the presence or absence of fibrous capsules was assessed using the same serial sections from the same blocks as used for CD68 immunohistochemistry. Representative pictures are provided in Additional file 1: Figure S1.

The semi-quantitative immunohistochemical grading of TILs and TAMs in tumors was determined by high-power microscopy (when there were more than five regions, the most abundant lymphocyte infiltration area was selected by the “hot spot” method) [22, 23]. Briefly, five fields with the richest infiltration of GC were selected from each slice, and the percentages of TILs and TAMs were calculated. The average of five fields was used as the density of TILs and TAMs [24]. First, a quantitative score based on the estimated percentage of immunopositive-stained cells among total cells was specified according to the following scale: 1 (< 1% cells); 2 (1–10% cells); 3 (11–33% cells); 4 (34–66% cells); and 5 (67–100% cells). Immunopositive cells were defined as those showing partial or complete staining within the cytoplasm and/or plasma membrane. Second, staining intensity was scored as follows: 0 (none), 1+ (mild), 2+ (moderate), and 3+ (intense). Finally, scores (ranging from 1 to 8) were calculated by adding the percentage positivity scores and the intensity scores for each section. The patient cohort was divided into two groups using the median value, with negative or positive CD8+ or CD68+ expression.

### Statistical analysis

Correlation between clinicopathological features and immunohistochemical variables was evaluated by the Chi-square test or Fisher exact test, as appropriate. The Kaplan-Meier method was employed to estimate the survival curve and the log-rank test for survival analysis. Important factors identified by univariate analysis were examined in multivariate analysis. A Cox proportional hazard model was applied for multivariate analysis to evaluate the independent effects of variables. The Harrell index of concordance (C-index), Akaike information criterion (AIC) [16], and Bayesian information criterion (BIC) [25] were calculated to compare the accuracy of the prognostic models. In addition, decision curve analysis was performed to determine the clinical utility of the prognostic model [26]. The statistical analyses were performed using SPSS 19.0 (SPSS Inc., Chicago, IL) and R version 3.1.2 (R Foundation for Statistical Computing, Vienna, Austria). Statistical significance was set at a 2-sided *p* < 0.05.

## Results

### Association of CD8+ TIL and CD68+ TAM with clinicopathological features

The patient clinicopathological features are summarized in Table 1. In this study, there were 401 patients [271 male (67.6%), 130 female (32.4%)]. Positive expression of CD8+ TIL and CD68+ TAM were 51.1% (205 out of 401) and 45.9% (184 out of 401) in our set, respectively. A CD8+ TIL-positive status negatively correlated with lymphovascular invasion (*p* = 0.044) and lymph node metastasis (*p* = 0.014), whereas such a correlation was not found in the CD68+ TAM-positive subgroup. Moreover, CD8+ TIL and CD68+ TAM did not significantly correlate with tumor size (*p* = 0.260, *p* = 0.441), tumor differentiation (*p* = 0.858, *p* = 0.840), tumor depth (*p* = 0.158, *p* = 0.225) or TNM stage (*p* = 0.123, *p* = 0.561). No significant correlations were found between expression of CD8+ TIL or CD68+ TAM and adjuvant chemotherapy. (Table 1).

**Table 1:** Correlation between TIL, TAM and clinicopathologic characteristics in the 401 gastric cancers

Characteristics	CD8+ TIL		*p* value	CD68+ TAM		*p* value
	Negative (%)	Positive (%)		Negative (%)	Positive (%)	
All cases	196 (48.6)	205 (51.1)		217 (54.1)	184 (45.9)	
Sex			0.596			
Female	61 (31.1)	69 (33.7)		72 (33.2)	58 (31.5)	0.749
Male	135 (68.9)	136 (68.3)		145 (66.8)	126 (68.5)	
Age (y)			0.915			0.668
<65	134 (68.4)	139 (67.8)		150 (69.1)	123 (66.8)	
≥ 65	62 (31.6)	66 (32.2)		67 (30.9)	61 (33.2)	
Tumor location			0.805			0.330
Proximal	58 (29.6)	59 (28.8)		68 (31.3)	49 (26.6)	
Middle	30 (15.3)	39 (19.0)		37 (17.1)	32 (17.4)	
Distal	96 (49.0)	95 (46.3)		103 (47.5)	88 (47.8)	
Entire	12 (6.1)	12 (5.9)		9 (4.1)	15 (8.2)	
Tumor size (cm)			0.260			0.441
<5	114 (58.2)	131 (63.9)		137 (63.1)	108 (58.7)	
≥ 5	82 (41.8)	74 (36.1)		80 (36.9)	76 (41.3)	
Differentiation			0.858			0.840
Differentiated	83 (42.3)	85 (41.5)		92 (42.4)	76 (41.3)	
Undifferentiated	113 (57.7)	120 (58.5)		125 (57.6)	108 (58.7)	
Lymphovascular invasion			0.044			0.130
Absent	101 (51.5)	127 (62.0)		131 (60.4)	97 (52.7)	
Present	95 (48.5)	78 (38.0)		86 (39.6)	87 (47.3)	
Tumor depth			0.158			0.225
T1/2	75 (38.3)	93 (45.4)		97 (44.7)	71 (38.6)	
T3/4	121 (61.7)	112 (54.6)		120 (55.3)	113 (61.4)	
Lymph node metastasis			0.014			0.919
Absent	65 (33.2)	93 (45.4)		86 (39.6)	72 (39.1)	
Present	131 (66.8)	112 (54.6)		131 (60.4)	112 (60.9)	
Pathological stage			0.123			0.561
I	57 (29.1)	78 (38.0)		78 (35.9)	57 (31.0)	
II	41 (20.9)	43 (21.0)		43 (19.8)	41 (22.3)	
III	98 (50.0)	84 (41.0)		96 (44.2)	86 (46.7)	
Adjuvant chemotherapy			0.222			0.917
Absent	65 (33.2)	80 (39.0)		79 (36.4)	66 (35.9)	
Present	131 (66.8)	125 (61.0)		138 (63.6)	118 (64.1)	

### Correlation between the CD8+ TIL/CD68+ TAM status and prognosis

Patients who were CD8+ TIL positive showed significantly improved RFS (Additional file 2: Figure S2A; *p* < 0.001) and OS (Additional file 2: Figure S2C; *p* < 0.001) compared with CD8+ TIL-negative patients. As show by Additional file 2: Figure S2B and Additional file 2: Figure S2D, the RFS and OS of the CD68+ TAM-positive group were significantly lower than that of the CD68+ TAM-negative group (both *p* < 0.001).

### Univariate and multivariate regression analysis

Lymphovascular involvement (*p* = 0.009), TNM stage (*p*<0.001), adjuvant chemotherapy (HR: 0.577, *p* = 0.039), positive CD8+ TIL status (HR: 0.372, *p* < 0.001) and positive CD68+ TAM status (*p*<0.001) were identified as independent prognostic factors that associated with RFS (Additional file 5: Table S1). Other factors including sex, age, tumor location, tumor size, tumor differentiation, were not significantly associated with patient recurrence-free survival. Similar results were obtained in the OS analysis (Additional file 5: Table S2).

### The combination of CD8+ TIL and CD68+ TAM statuses

**Fig. 1:**
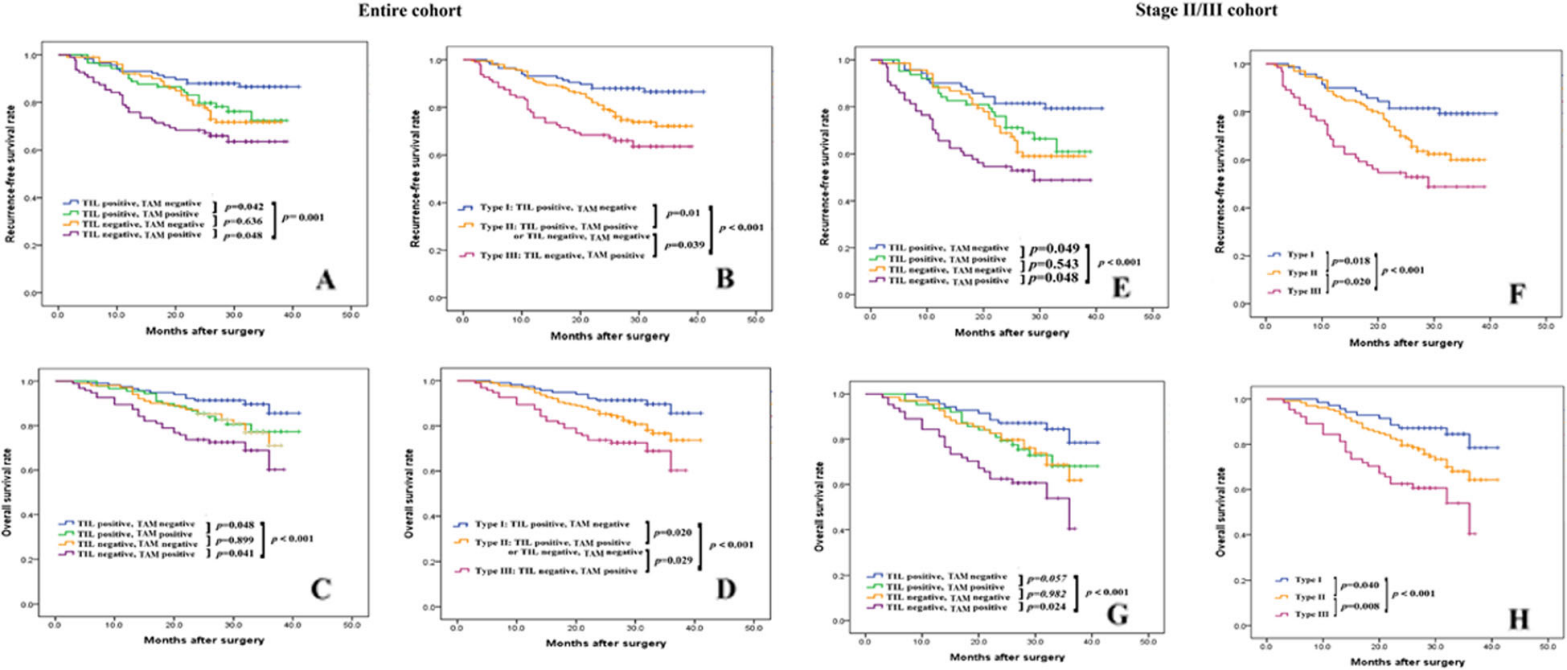
Kaplan-Meier curves for RFS and OS, according to TIL and TAM statuses in the entire cohort (**a** and **c**) and in the stage II/III cohort (**e** and **g**). Type I is TIL positive and TAM negative; type II is TIL positive and TAM positive or TIL negative and TAM negative, and type III is TIL negative and TAM positive (**b**, **d**, **f** and **h**)

We classified the gastric cancer cases into 4 groups based on their CD8+ TIL and CD68+ TAM statuses (Fig. 1). The proportion of the patients in groups 1, 2, 3, and 4 was 28.9% (*n* = 116), 22.2% (*n* = 89), 25.2% (*n* = 101), and 23.7% (*n* = 95), respectively. Interestingly, we found no significant differences in RFS and OS between group 2 and group 3 (*p* = 0.633, *p* = 0.899, respectively, Fig. 1a, c). Thus, we defined group 1 as type I, groups 2 and 3 as type II, and group 4 as type III (Fig. 1b, d). Additional stratified analysis showed significant differences in RFS and OS among these 3 types in the stage II/III cohort (Fig. 1e-h). However, no differences were observed in RFS and OS among these 3 types for stage I disease due to the excellent prognosis of stage I patients (data not shown).

### Extension of the TNM staging system to include CD8+ TIL/CD68+ TAM status

When analyzing RFS, the C-index was 0.7699, when assessed only with TNM stage, and the outcomes improved to 0.8126, when CD8+ TIL/CD68+ TAM status was added. Also in the RFS analysis, the AIC and BIC were 1009.875 and 1013.869, respectively, when assessed only with TNM stage, and the outcomes decreased to 998.756 and 1001.750, respectively, when CD8+ TIL/CD68+ TAM status was added (Table 2). Similar results were obtained from OS analysis which corroborated the results from the RFS analysis (Table 2).

**Table 2:** Comparison of the prognostic accuracies of TNM staging system and tumour-infiltrating immune cells

RFS	Model			*p* value
	TNM	TIL + TAM	TNM + (TIL + TAM)	
C-index (95% CI)	0.7699 (0.7159–0.8013)	0.6055 (0.5538–0.6782)	0.8126 (0.7791–0.8504)	<0.001
AIC	1009.875	1104.745	998.756	**/**
BIC	1013.869	1108.739	1001.750	**/**
OS				
C-index (95% CI)	0.7428 (0.7053–0.7932)	0.6329 (0.5881–0.7011)	0.8030 (0.7626–0.8485)	<0.001
AIC	825.9906	880.5326	815.0896	**/**
BIC	829.9846	884.5266	820.0835	**/**

C-index indicates Harrell concordance index; *AIC* indicates Akaike Information Criterion, *BIC* indicates Bayesian Information Criterion, *AUC* indicates area under the curve

### Assessment of the clinical utility of the prognostic model with CD8+ TIL/CD68+ TAM status using decision curve analysis

Next, we assessed the clinical application of the prognostic model that included CD8+ TIL/CD68+ TAM status. The results indicate that the constructed model is advantageous with a higher threshold probability and improved performance for predicting 3-year RFS and 3-year OS than the TNM stage or CD8+ TIL/CD68+ TAM status alone (Additional file 3: Figure S3).

### Associations between postoperative adjuvant chemotherapy (PAC) and the CD8+ TIL/CD68+ TAM status

**Fig. 2:**
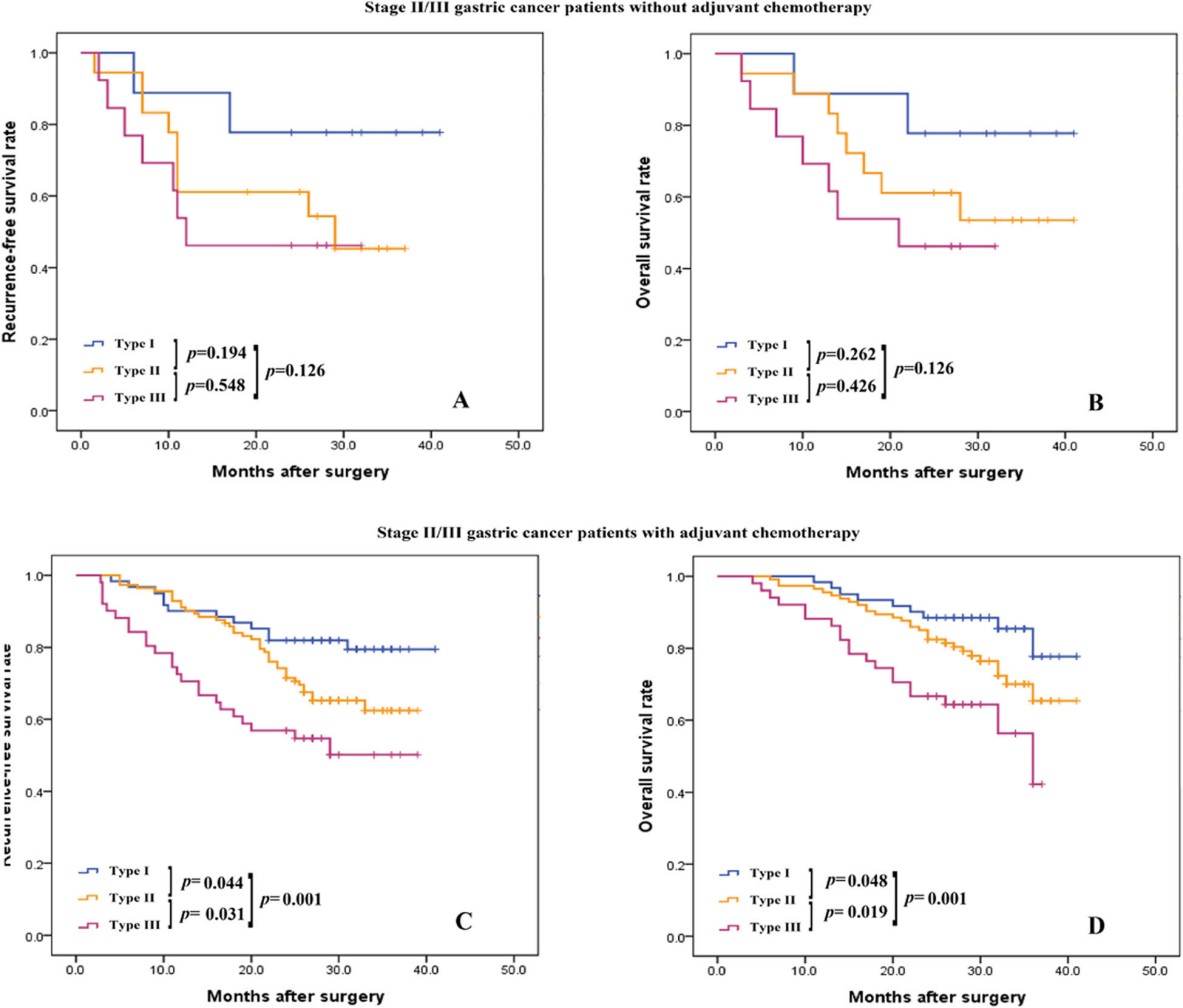
Kaplan-Meier analysis of RFS and OS in stage II/III patients received postsurgical adjuvant chemotherapy (PAC) according to TIL/TAM status. Patients who did not receive PAC show no prognostic significance in RFS (**a**, *p* = 0.126), and OS (**b**, *p* = 0.126) according to TIL/TAM status, respectively. Whereas, patients with stage II/III received 5-FU-based PAC positively correlated with RFS (**c**, *p* = 0.001) and OS (**d**, *p* = 0.001) according to TIL/TAM status, respectively

For the entire cohort of patients who did not receive PAC treatment, the CD8+ TIL/CD68+ TAM status was not associated with OS (Additional file 4: Figure S4B; *p* = 0.085). In contrast, for stage II/III patients who did not receive PAC, the CD8+ TIL/CD68+ TAM status was not associated with RFS or OS (Fig. 2a; *p* = 0.126 and Fig. 2b, *p* = 0.126, respectively). Importantly, both in the entire cohort and in stage II/III patients who received PAC, those that were classified as type I had better RFS and OS than those classified as type II or III (Additional file 4: Figure S4C, *p*<0.001, Additional file 4: Figure S4D, *p*<0.001; and Fig. 2c, *p* = 0.001, Fig. 2D, *p* = 0.001, respectively). Patients who received PAC and were CD8+ TIL positive and CD68+ TAM negative had a reduced risk of short survival, both in the entire cohort and in the subgroup with stage II/III disease (HR: 0.466, 95% CI: 0.265–0.821, *p* = 0.008; HR: 0.458, 95% CI: 0.257–0.817, *p* = 0.008, respectively), whereas patients who did not receive PAC did not show the same risk benefit (Table 3). Altogether, these results suggest that gastric cancer patients with a CD8+ TIL-positive and CD68+ TAM-negative status (defined as type I above) might benefit more from PAC.

**Table 3:** Hazard ratios for risk of mortality in gastric cancer patients receiving postoperative adjuvant chemotherapy (pac) or not according to TIL and TAM status

		Risk of mortality		
	Variable	No. of patients	(TIL positive + TAM negative) vs. others	
Entire cohort		401 (100%)	HR (95% CI)	*p* value
	With PAC	256 (63.8%)	0.466 (0.265–0.821)	0.008
	Without PAC	145 (36.2%)	0.543 (0.195–1.515)	0.243
Stage II/III cohort		266 (100%)		
	With PAC	226 (85.0%)	0.458 (0.257–0.817)	0.008
	Without PAC	40 (15.0%)	0.534 (0.191–1.496)	0.233

## Discussion

GC is an inflammation-related cancer characterized by a large degree of multinuclear and monocyte infiltration, including lymphocytes and macrophages [16, 27]. Tumor-associated immune cells affect complex microenvironments, with prognostic value in previous studies [11, 16, 28]. To date, there are several markers for TILs, such as CD3, CD4, CD8, Foxp3, and Granzyme B [29]. In the present study, we stained for CD8 on T cells, as reported [24, 30]. TAMs can be classified into two phenotypes: M1 (classically activated macrophages) and M2 (alternatively activated macrophages) [31, 32], and using immunochemistry, TAMs can be assessed by anti-CD68, M1-type TAMs by anti-HLA-DR and M2-type TAMs by anti-CD163 [32, 33]. However, it is largely unclear which macrophages have the greatest impact on the efficacy of chemotherapy for GC [34]. CD68 has been widely accepted as a specific marker of TAMs in human cancer [35, 36]. A recent report found high expression of CD68+ TAMs to be associated with recurrence of melanoma [36], and other studies have reported the clinical and functional significance of CD68+ TAMs in GC, though without addressing the M1 and M2 subsets [37, 38] . Thus, we also used CD68 as a marker of TAMs in this study.

Previous studies have explored the relationship between TIL infiltration and GC outcomes, but these studies have produced different results [39, 40]. For example, Fukuda et al. [39] found no significant difference in survival rates among patients with significant or slight TIL infiltration. In contrast, Lee et al. reported that the OS of GC patients with a high CD8+ TIL density tended to be longer than that of patients with a low TIL density at the same TNM stage, which was consistent with our findings [40]. In our study, the RFS and OS of TIL-positive cases were significantly better than those of TIL-negative cases. These conflicting results may be caused by different methods used to determine TIL strength. In addition, the function of intratumoral CD8+ lymphocytes in tumors is interlinked with other immune cells, and accumulating evidence suggests that TAMs plays an important role in cancer progression [41–43]. However, conflicting prognostic data have been reported [33]. Ishigami et al. [44] observed a negative correlation between TAMs and the prognosis of GC patients, whereas Ohno et al. [45] concluded that the aggregation of TAMs within the tumor nest had a beneficial effect. Zhang et al. [46] also evaluated TAMs in 180 GC patients and found no significant correlation between these cells and OS. In the present study, CD68+ TAMs were found to be an independent risk factor for a worse GC prognosis.

Indeed, it is difficult to characterize complex tumor microenvironment using a single immune marker [43]. Nakanishi et al. reported that the ratio of CD68+ macrophages/CD57+ cells was closely related to prognosis of renal cell carcinoma [47]. Therefore, the main purpose of this study was to explore the effect of a combination of TILs and TAMs on the prognosis and efficacy of chemotherapy for GC, rather than the effect of different types of macrophages on prognosis. In fact, the use of multiparametric analysis to characterize immune cell types, such as CD8+ TILs and CD68+ TAMs, in the present study may provide a more comprehensive picture of the immune phenotypes within the TME, which may help in stratifying patients with the best chance of responding to immunotherapy. Using the CD8+ TIL/CD68+ TAM status, we were able to classify samples into 3 types (Type 1: CD8+ TIL positive and CD68+ TAM negative; Type 2: CD8+ TIL positive and CD68+ TAM positive or CD8+ TIL negative and CD68+ TAM negative; Type 3: CD8+ TIL negative and CD68+ TAM positive).

Traditional prognostic models for GC patients depend on the TNM staging system, which is derived from biological phenotypes centered on cancer cells. In fact, even patients with the same stage of cancer may have very different prognoses. In this study, we first found that incorporating CD8+ TILs/CD68+ TAMs into the current TNM staging system may increase prognostic value, thereby better identifying patients with different prognoses and providing better risk-oriented treatment. In general, incorporating molecular biomarkers or biologically driven classification into staging systems may better predict prognosis and treatment options and promote the development of personalized or precise medicine [48, 49]. TILs and TAMs are key in tumor-related inflammation and binding with other immune cells, which are processes considered to be evidence of host-growth interaction with tumors [31, 33, 50]. Previously, Zhang et al. generated a nomogram integrating TAMs, tumor T stage, N stage, and distant metastasis to predict the OS rate of GC [46]. In this study, we generated a novel and powerful staging system for GC patients based on TIL/TAM-based immune status and the tumor cell-centered TNM system. Our results suggest that incorporating the TIL/TAM status into the established TNM system can more accurately quantify prognostic risk.

Patients with stage II or III GC are considered to be candidates for PAC. However, it is uncertain whether all patients need PAC, as a large proportion of them do not appear to benefit from this approach. Therefore, the development of an improved PAC benefit prediction model has gained much interest [6]. Indeed, there is much evidence that the success of anticancer therapies, including traditional cytotoxic compounds, radiotherapy and targeted drugs, depends, at least in part, on activation of the anticancer immune response [51]. As previously described, CD68+ TAMs increase the response of many malignant tumors (such as stage III colorectal cancer) to 5-FU adjuvant therapy [52]. In this study, we further assessed the relationship between TILs/TAMs and survival in a subgroup of stage II/III patients receiving PAC. The results indicated that among patients receiving PAC, those with simultaneously high TIL and low TAM infiltration were more likely to have increased RFS and OS rates compared with the other 3 groups, indicating that CD8+ TILs/CD68+ TAMs may be an important factor in predicting the effect of chemotherapy. Immunotherapy, including immune checkpoint blockade, has been considered an important component of anti-cancer therapy [53, 54], and the TIL/TAM status might be an interesting target of investigation for the immunotherapy options that are emerging in this setting. A recent study found that PD-L1 is not only expressed in cancer cells but also in TILs at a high rate [55]. Furthermore, Harada et al. reported that TAMs are highly associated with PD-L1 expression in GC cells, suggesting that macrophage infiltration is a potential therapeutic target [56]. The authors also indicated that CSF1/CSF1R blockade might reduce PD-L1 expression in tumor cells [56]. Clinically, the status of CD8+ TILs/ CD68+ TAMs is helpful for stratifying patients with stage II/III GC receiving PAC, and prognostic stratification based on the TIL/TAM status can guide doctors in adopting tailor-made treatment plans according to patient performance. For example, strengthening a 5-FU-based treatment regimen or adding macrophage-targeted therapies (such as CSF1/CSF1R and/or PD-L1 inhibitors) is a potential strategy for type II or III GC with good performance. Currently, a phase 1, open-label, global study is underway to assess the combination of CSF1R antagonists and checkpoint blockades (NCT0323191). These results may help clinicians better select patients who need more aggressive adjuvant therapy or more in-depth follow-up, even though these patients might not be considered to be at high risk according to traditional clinicopathological features.

This study has some limitations. First, the study was retrospective. Although all parameters of prospective clinical trials were included, the number of patients receiving PAC was relatively small, and the prognostic significance of the PAC regimen and PAC cycle was not compared. Second, as a currently unresolved issue [24, 57], the specimens used and semi-quantitative immunohistochemical evaluation may not fully reflect the status of the tumor immune microenvironment. TAMs are more diverse and heterogeneous than cells with single markers or M1/M2 phenotypes. Further studies are needed to determine the interaction between different TAM and cancer outcomes. Third, the follow-up time was relatively short. Overall, in-depth in vitro and in vivo experiments are urgently needed to provide mechanical insight.

## Conclusions

The TIL/TAM status is a promising biomarker for predicting the prognosis of GC. Our model incorporates immune parameters into the established TNM staging system to help clinicians and patients quantify the benefits of adjuvant chemotherapy after GC resection and formulate personalized treatment recommendations and treatment decisions. However, these results should be validated by a large, prospective, multiagency study.

## Additional files

Additional file 1: **Figure S1.** TIL and TAM status in gastric cancer. A, TIL status in the invasive margin of tumors. Original magnification, 200× (left panels). B, TAM status in the invasive margin of tumors. Original magnification, 200× (right panels). (TIF 331 kb)

Additional file 2: **Figure S2.** Kaplan–Meier curves for RFS and OS of gastric cancer patients according TIL (A and C) or TAM (B and D) status. (TIF 271 kb)

Additional file 3: **Figure S3.** The TNM staging system combined with TIL/TAM status was compared to the TNM staging system alone and TIL/TAM status alone in terms of 3-year RFS and OS (A and B). Using decision curve analysis, the TNM staging system combined with TIL/TAM status showed superior net benefit compared to the TNM staging system alone and TIL/TAM status alone. (TIFF 304 kb)

Additional file 4: **Figure S4.** Kaplan-Meier analysis of RFS and OS in entire cohort patients received postsurgical adjuvant chemotherapy (PAC) according to TIL/TAM status. Patients who did not receive PAC show prognostic significance in RFS (A, *p* = 0.046), but no prognostic significance in OS (B, *p* = 0.085) according to TIL/TAM status, respectively. Whereas, patients received 5-FU-based PAC positively correlated with RFS (C, *p*<0.001) and OS (D, *p*<0.001) according to TIL/TAM status, respectively. (TIF 346 kb)

Additional file 5: **Table S1.** Univariate and Multivariable Analysis of Recurrence-free Survival in 401 Patients With Gastric Cancer. **Table S2.** Univariate and Multivariable Analysis of Overall Survival in 401 Patients With Gastric Cancer. (DOCX 21 kb)

## Data Availability

The datasets used and/or analyzed during the current study are available from the corresponding author on reasonable request.

## Abbreviations

CD68: Cluster of differentiation 68
CD8: Cluster of differentiation 8
GC: Gastric cancer
OS: Overall survival
PAC: Postoperative adjuvant chemotherapy
RFS: Recurrence-free survival
TAM: Tumor-associated macrophages
TIL: Tumor-infiltrating lymphocytes
