# Supplementary figures and images for: Tumor-infiltrating CD8+ T cells combined with tumor-associated CD68+ macrophages predict postoperative prognosis and adjuvant chemotherapy benefit in resected gastric cancer
Source: BMC Cancer. 2019 Sep 14;19:920. doi: 10.1186/s12885-019-6089-z (PMC6744628; doi:10.1186/s12885-019-6089-z)

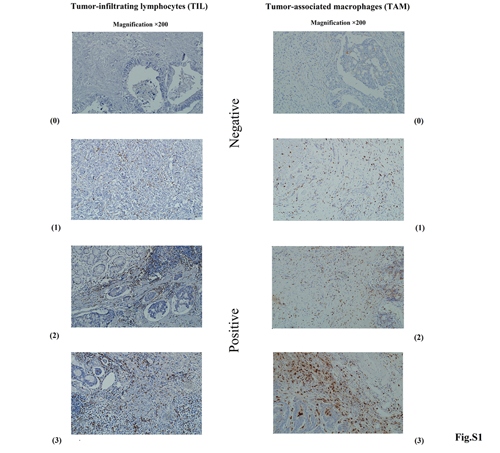

Supplement: Supplementary file 1 — Figure S1. TIL and TAM status in gastric cancer. A, TIL status in the invasive margin of tumors. Original magnification, 200× (left panels). B, TAM status in the invasive margin of tumors. Original magnification, 200× (right panels). (TIF 331 kb) [file 12885_2019_6089_MOESM1_ESM.tif]

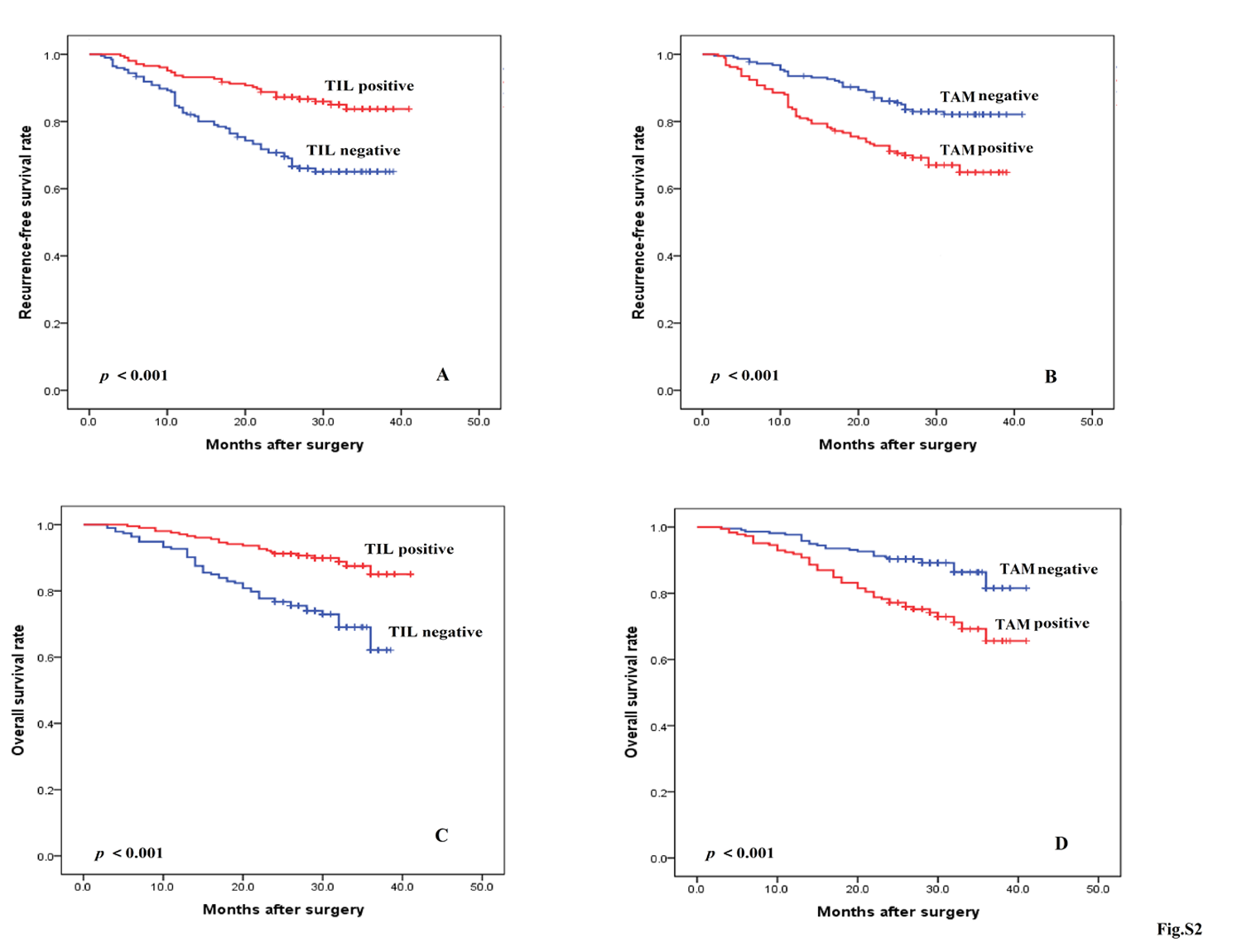

Supplement: Supplementary file 2 — Figure S2. Kaplan–Meier curves for RFS and OS of gastric cancer patients according TIL (A and C) or TAM (B and D) status. (TIF 271 kb) [file 12885_2019_6089_MOESM2_ESM.tif]

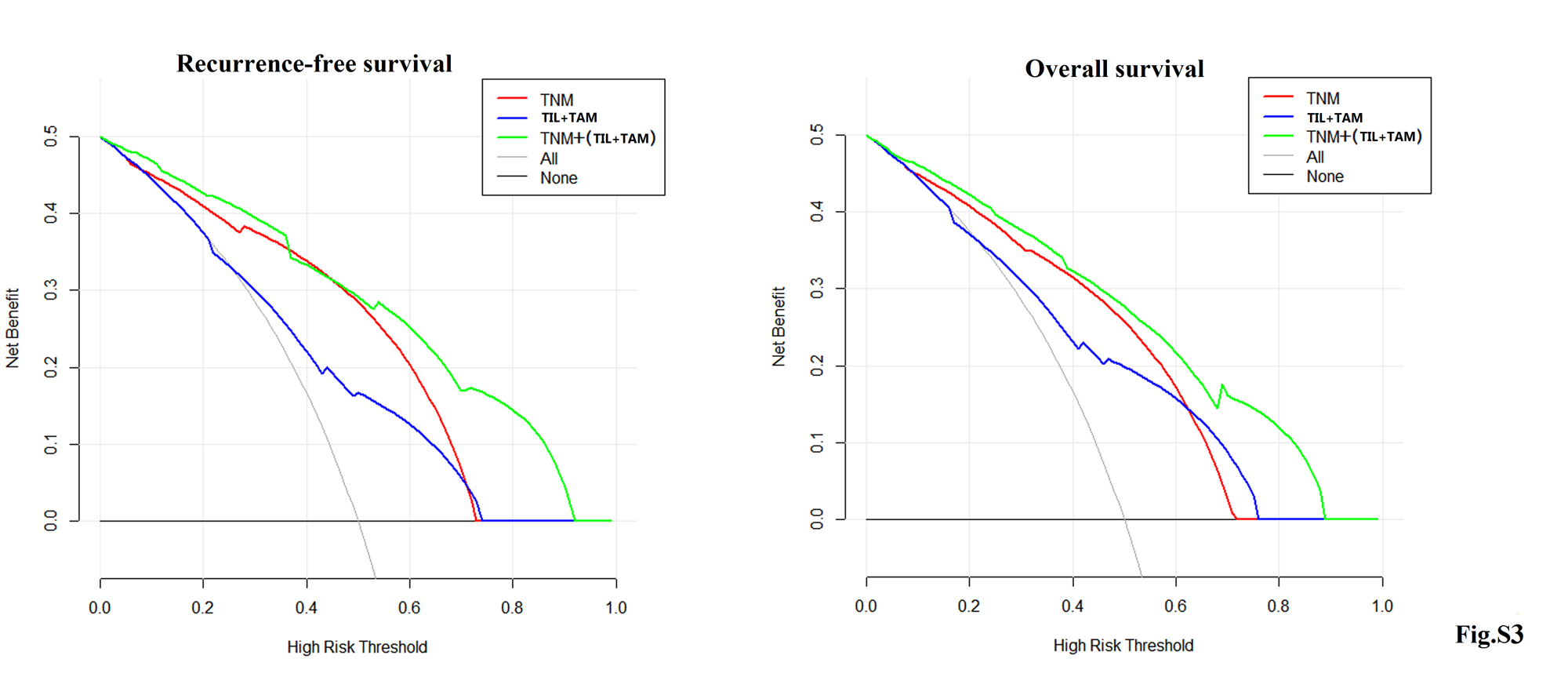

Supplement: Supplementary file 3 — Figure S3. The TNM staging system combined with TIL/TAM status was compared to the TNM staging system alone and TIL/TAM status alone in terms of 3-year RFS and OS (A and B). Using decision curve analysis, the TNM staging system combined with TIL/TAM status showed superior net benefit compared to the TNM staging system alone and TIL/TAM status alone. (TIFF 304 kb) [file 12885_2019_6089_MOESM3_ESM.tiff]

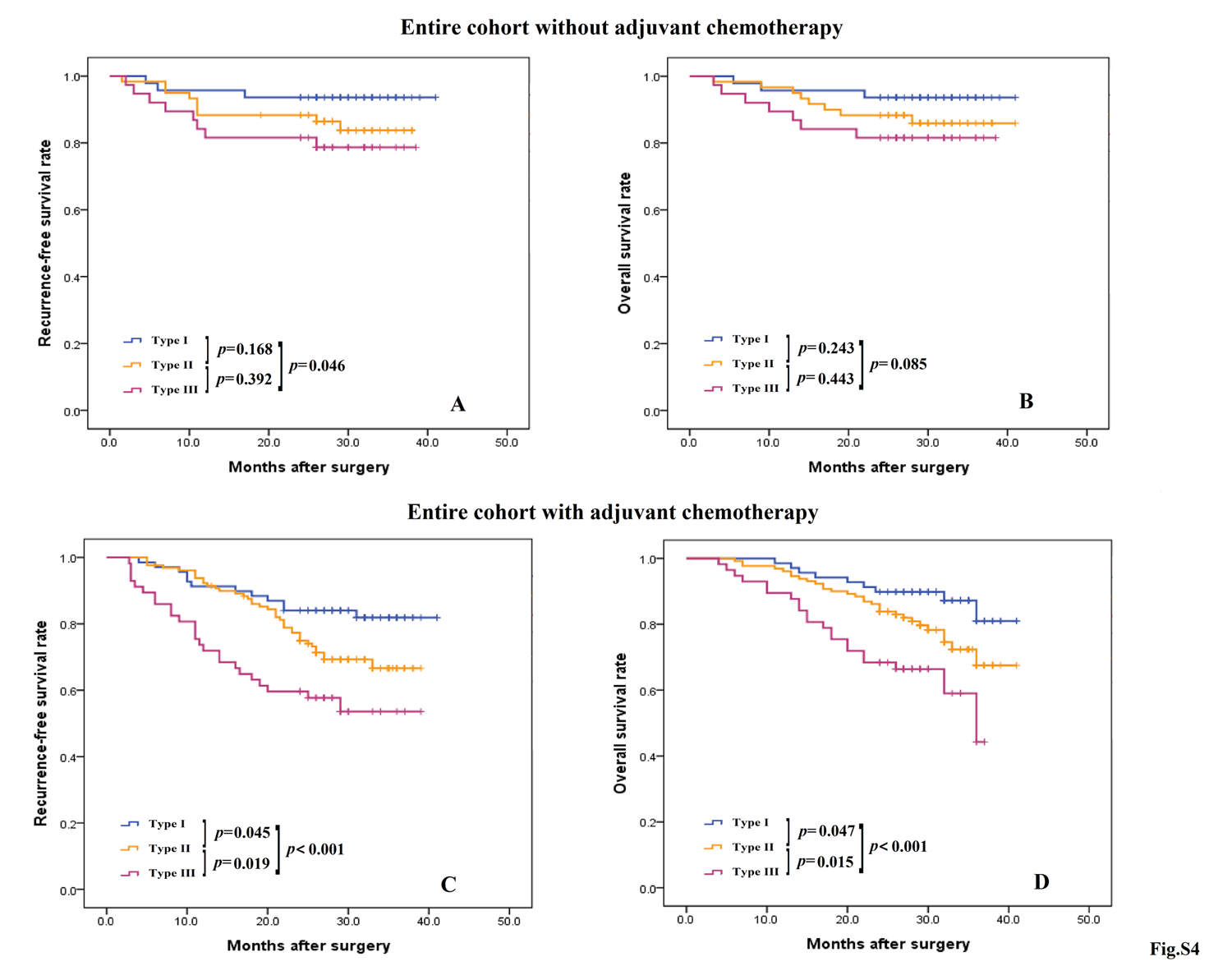

Supplement: Supplementary file 4 — Figure S4. Kaplan-Meier analysis of RFS and OS in entire cohort patients received postsurgical adjuvant chemotherapy (PAC) according to TIL/TAM status. Patients who did not receive PAC show prognostic significance in RFS (A, p = 0.046), but no prognostic significance in OS (B, p = 0.085) according to TIL/TAM status, respectively. Whereas, patients received 5-FU-based PAC positively correlated with RFS (C, p<0.001) and OS (D, p<0.001) according to TIL/TAM status, respectively. (TIF 346 kb) [file 12885_2019_6089_MOESM4_ESM.tif]
