# Supplementary material for: Tumor-infiltrating CD8+ T cells combined with tumor-associated CD68+ macrophages predict postoperative prognosis and adjuvant chemotherapy benefit in resected gastric cancer
Source: BMC Cancer. 2019 Sep 14;19:920. doi: 10.1186/s12885-019-6089-z (PMC6744628; doi:10.1186/s12885-019-6089-z)
Supplement: Supplementary file 5 — Table S1. Univariate and Multivariable Analysis of Recurrence-free Survival in 401 Patients With Gastric Cancer. Table S2. Univariate and Multivariable Analysis of Overall Survival in 401 Patients With Gastric Cancer. (DOCX 21 kb) [file 12885_2019_6089_MOESM5_ESM.docx]

**Table S1. Univariate and Multivariable Analysis of** **Recurrence-free Survival in 401 Patients With Gastric Cancer**

|  | **Univariate** | | | **Multivariable** | | |
| --- | --- | --- | --- | --- | --- | --- |
| **Variables** | Hazard Ratio | 95% CI | p value | Hazard Ratio | 95% CI | *p* value |
| Sex (male) | 1.246 | 0.779-1.945 | 0.332 |  |  |  |
| Age (≥65) | 1.182 | 0.776-1.801 | 0.436 |  |  |  |
| Tumor location (lower third) | 0.807 | 0.655-0.994 | **0.044** | 0.985 | 0.806-1.205 | 0.884 |
| Tumor size (≥5cm) | 3.792 | 2.480-5.800 | **＜0.001** | 1.283 | 0.818-2.010 | 0.278 |
| Tumor differentiation (undifferentiated) | 2.130 | 1.357-3.341 | **0.001** | 1.230 | 0.764-1.979 | 0.395 |
| Lymphovascular involvement | 6.354 | 3.877-10.416 | **＜0.001** | 2.028 | 1.195-3.444 | **0.009** |
| Pathological stage (stage III) | 6.547 | 3.910-10.964 | **＜0.001** | 5.439 | 3.079-9.609 | **＜0.001** |
| Adjuvant chemotherapy | 0.435 | 0.266-0.712 | **0.001** | 0.577 | 0.342-0.973 | **0.039** |
| Positive CD8+ TIL | 0.375 | 0.243-0.578 | **＜0.001** | 0.372 | 0.239-0.579 | **＜0.001** |
| Positive CD68+ TAM | 2.150 | 1.424-3.245 | **＜0.001** | 2.182 | 1.435-3.318 | **＜0.001** |

**Table S2. Univariate and Multivariable Analysis of Overall Survival in 401 Patients With Gastric Cancer**

|  | **Univariate** | | | **Multivariable** | | |
| --- | --- | --- | --- | --- | --- | --- |
| **Variables** | Hazard Ratio | 95% CI | p value | Hazard Ratio | 95% CI | *p* value |
| Sex (male) | 1.053 | 0.065-1.704 | 0.832 |  |  |  |
| Age (≥65) | 1.396 | 0.884-2.203 | 0.152 |  |  |  |
| Tumor location (lower third) | 0.924 | 0.738-1.156 | 0.488 |  |  |  |
| Tumor size (≥5cm) | 3.581 | 2.236-5.736 | **＜0.001** | 1.482 | 0.894-2.458 | 0.127 |
| Tumor differentiation (undifferentiated) | 2.115 | 1.282-3.489 | **0.003** | 1.295 | 0.769-2.182 | 0.331 |
| Lymphatic involvement | 4.438 | 2.774-7.100 | **＜0.001** | 2.309 | 1.237-4.310 | **0.007** |
| Pathological stage (stage III) | 6.368 | 3.624-11.189 | **＜0.001** | 3.216 | 1.850-5.591 | **＜0.001** |
| Adjuvant chemotherapy | 0.518 | 0.306-878 | **0.014** | 0.569 | 0.326-0.994 | **0.048** |
| Positive CD8+ TIL | 0.343 | 0.211-559 | **＜0.001** | 0.367 | 0.224-0.603 | **＜0.001** |
| Positive CD68+ TAM | 2.463 | 1.548-3.918 | **＜0.001** | 2.466 | 1.538-3.954 | **＜0.001** |
